# Analysis of epistatic interactions and fitness landscapes using a new geometric approach

**DOI:** 10.1186/1471-2148-7-60

**Published:** 2007-04-13

**Authors:** Niko Beerenwinkel, Lior Pachter, Bernd Sturmfels, Santiago F Elena, Richard E Lenski

**Affiliations:** 1 Program for Evolutionary Dynamics, Harvard University, Cambridge, MA 02138, USA; 2 Department of Mathematics, University of California, Berkeley, CA 94720, USA; 3 Instituto de Biología Molecular y Celular de Plantas, Consejo Superior de Investigaciones Científicas-UPV, 46022 València, Spain; 4 Department of Microbiology and Molecular Genetics, Michigan State University, East Lansing, MI 48824, USA

## Abstract

**Background:**

Understanding interactions between mutations and how they affect fitness is a central problem in evolutionary biology that bears on such fundamental issues as the structure of fitness landscapes and the evolution of sex. To date, analyses of fitness landscapes have focused either on the overall directional curvature of the fitness landscape or on the distribution of pairwise interactions. In this paper, we propose and employ a new mathematical approach that allows a more complete description of multi-way interactions and provides new insights into the structure of fitness landscapes.

**Results:**

We apply the mathematical theory of gene interactions developed by Beerenwinkel et al. to a fitness landscape for *Escherichia coli *obtained by Elena and Lenski. The genotypes were constructed by introducing nine mutations into a wild-type strain and constructing a restricted set of 27 double mutants. Despite the absence of mutants higher than second order, our analysis of this genotypic space points to previously unappreciated gene interactions, in addition to the standard pairwise epistasis. Our analysis confirms Elena and Lenski's inference that the fitness landscape is complex, so that an overall measure of curvature obscures a diversity of interaction types. We also demonstrate that some mutations contribute disproportionately to this complexity. In particular, some mutations are systematically better than others at mixing with other mutations. We also find a strong correlation between epistasis and the average fitness loss caused by deleterious mutations. In particular, the epistatic deviations from multiplicative expectations tend toward more positive values in the context of more deleterious mutations, emphasizing that pairwise epistasis is a local property of the fitness landscape. Finally, we determine the geometry of the fitness landscape, which reflects many of these biologically interesting features.

**Conclusion:**

A full description of complex fitness landscapes requires more information than the average curvature or the distribution of independent pairwise interactions. We have proposed a mathematical approach that, in principle, allows a complete description and, in practice, can suggest new insights into the structure of real fitness landscapes. Our analysis emphasizes the value of non-independent genotypes for these inferences.

## Background

Understanding the structures of fitness landscapes is central to evolutionary biology. The image of populations evolving on fitness landscapes traces to Sewall Wright's seminal work in the thirties [3]. Since then, several types of fitness landscapes have been discussed in the literature [3-8], resulting in some confusion. The surface of a landscape may represent the relative fitness of individual types, or the average fitness of a population. In the former case, the underlying coordinates describe either the genotypic or phenotypic state of an individual; in the latter case, the coordinates describe either gene frequencies or average phenotypes in a population. Our paper concerns the mathematical analysis and interpretation of fitness landscapes where the height of the surface represents the relative fitness of individuals and the coordinates are different genotypes. In this evolutionary context, fitness measures the expected reproductive success of an individual having a specific genotype in some particular environment. Thus, a fitness landscape is given by assigning to each genotype *g *its fitness *w*_*g*_.

If all mutations were strictly additive or multiplicative in their effects on fitness, then it would be rather easy to describe the structure of fitness landscapes and understand the resulting dynamics of adaptation by natural selection. However, many mutations interact with one another in complex ways. For example, two or more mutations may interact such that their combined effect on fitness is much greater or much less than predicted from their individual effects; their combined effect may even be opposite in sign to the expectation based on their individual effects. These deviations from simple expectations are called epistasis [8,9], and they determine the shape of fitness landscapes, including curvature and ruggedness. Understanding the prevalence and mathematical forms of these epistatic interactions is important for many issues in evolutionary biology including the dynamics of adaptation and divergence [3,8,10-12], reproductive isolation and speciation [8,12-16], the evolution of sexual reproduction [17-20], the robustness of organisms to developmental, environmental, and mutational perturbations [21-24], the persistence of drug-resistant pathogens [25,26], and more.

Over the last decade, several studies have sought to examine the form and prevalence of epistatic interactions by measuring the fitness effects of numerous mutations alone and in combination in viruses, bacteria, fungi, and animals [2,27-31]. To date, these analyses have focused on the overall directional curvature of fitness as a function of the number of mutations, on the distribution of pairwise interactions, or on both. In this paper, we introduce a new mathematical approach that allows a more complete description of multi-locus interactions in a fitness landscape. The benefits of this approach become evident from our analysis of a fitness landscape of *E. coli *that was obtained and first analyzed by Elena and Lenski [2]. They measured relative fitness values for 37 genotypes at 9 loci including the non-mutated parental strain or "wild-type", 9 single mutants, and 27 double mutants that each had a different pair of mutations. Although there are 36 (= 9·8/2) possible double mutants, only 27 were constructed due to limitations of the markers used for strain construction. No triple mutants or higher-order combinations were constructed in this experiment.

We organized the 37 genotypes in the symmetric 10-by-10 matrix with missing values as shown in Table 1. For clarity, we renamed the 9 mutations in the original study as (*a*, *b*, *c*), (*r*, *s*, *t*), and (*x*, *y*, *z*), where all genotypes in each of the three groups share the same antibiotic-resistance markers. Notice that double mutants, each denoted by a pair of letters, were produced except for those consisting of pairs from the same set of three adjacent letters. For example, *a *was paired with *r*, s, *t*, *x*, *y*, and *z*, but not with either *b *or *c*. Table 2 reports, for each genotype in the corresponding cell of Table 1, the median fitness value obtained from ten replicate assays by Elena and Lenski [2].

**Table 1:** Genotype space.

*w*	*a*	*b*	*c*	*r*	*s*	*t*	*x*	*y*	*z*
*a*				*ar*	*as*	*at*	*ax*	*ay*	*az*
*b*				*br*	*bs*	*bt*	*bx*	*by*	*bz*
*c*				*cr*	*cs*	*ct*	*cx*	*cy*	*cz*
*r*	*ar*	*br*	*cr*				*rx*	*ry*	*rz*
*s*	*as*	*bs*	*cs*				*sx*	*sy*	*sz*
*t*	*at*	*bt*	*ct*				*tx*	*ty*	*tz*
*x*	*ax*	*bx*	*cx*	*rx*	*sx*	*tx*			
*y*	*ay*	*by*	*cy*	*ry*	*sy*	*ty*			
*z*	*az*	*bz*	*cz*	*rz*	*sz*	*tz*			

All 37 *E. coli *genotypes used in this study are listed in the matrix. The wild-type strain is denoted *w*, the single mutants are denoted by the letters *a*, *b*, *c*, *r*, *s*, *t*, *x*, *y*, *z*, and the double mutants by pairs of these letters. Owing to the symmetry of the matrix, all genotypes different from the wild-type are shown twice. Genotypes along the diagonal do not exist. The other empty cells, such as *ab*, correspond to double mutants that are missing owing to the specific experimental design; no triple mutants were constructed in this experiment.

**Table 2:** Fitness data.

1.000	0.976	0.708	0.975	0.981	0.984	0.995	0.978	0.564	0.593
0.976				0.990	0.973	0.990	0.982	0.718	0.500
0.708				0.964	0.684	0.694	0.782	0.664	0.510
0.975				0.983	0.975	0.974	0.977	0.650	0.482
0.981	0.990	0.964	0.983				0.718	0.988	0.524
0.984	0.973	0.684	0.975				0.724	0.986	0.490
0.995	0.990	0.694	0.974				0.982	0.679	0.508
0.978	0.982	0.782	0.977	0.718	0.724	0.982			
0.564	0.718	0.664	0.650	0.988	0.986	0.679			
0.593	0.500	0.510	0.482	0.524	0.490	0.508			

Each entry shows the fitness value of the corresponding genotype in Table 1. Fitness is reported as the median of ten replicate assays performed to estimate the growth-rate advantage of the respective mutant genotype relative to the wild-type strain [2]. Values above and below the diagonal are the same. Genotypes along the diagonal do not exist; other empty cells correspond to genotypes that are missing owing to the specific experimental design.

**Figure 1:**
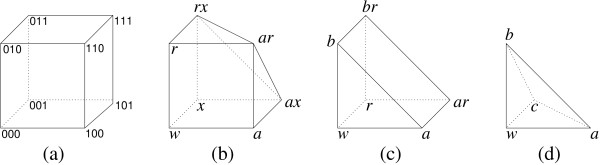
**Three-dimensional genotopes**. The genotope of the complete bi-allelic three-locus system with eight genotypes is the regular cube, depicted in (a). The three-locus systems that arise from data structured as in Table 1 are displayed in (b), (c), and (d). The genotope (b) lacks the triple mutant, genotope (c) lacks the triple and one double mutant, and genotope (d) contains only the wild-type and the single mutants.

The experimental design is illustrated geometrically in Figure 1, which shows the *genotopes *of all three-locus subsystems of the nine-locus system. A genotope is the set of all possible allele frequencies for a collection of genotypes. As a reference point, Figure 1a shows the regular cube representing all possible allele frequencies for the complete bi-allelic three-locus system. The absence of triple mutants and some of the double mutants from the present dataset gives rise to genotopes that are subsets of the cube. Each three-locus subsystem is determined by choosing three of the nine mutations, and there are three distinct types. First, choosing all three mutations from different groups, for example *a*, *r*, and *x*, induces the genotope in Figure 1b, which is obtained by slicing off the missing triple mutant from the cube. Second, choosing exactly two mutations from the same group, for example *a*, *b*, and *r*, further prunes the cube to a triangular prism as in Figure 1c. Finally, choosing all three mutations from the same group, for example *a*, *b*, and *c*, results in a tetrahedron such as the one shown in Figure 1d.

The goal of our analysis is to describe the geometry of the *E. coli *fitness landscape obtained by Elena and Lenski [2]. The fitness of a genotype was measured as the rate of population growth, expressed relative to the growth rate for the wild-type. These authors calculated pairwise interactions by considering the 27 equations of the following sort: *w·ar - a·r*, where *w *is the fitness of the wild-type, *ar *is the fitness of a double mutant, and *a *and *r *are fitness values for each associated single mutant. They found many significant deviations from zero, including several cases of both positive and negative epistasis. The key messages of our paper are that additional types of gene interactions exist even within this fitness landscape, and that geometric methods can be used to describe and analyze the system more exhaustively.

Our analysis of this landscape is based on the approach developed by Beerenwinkel et al. [1]. We identify a comprehensive set of 243 gene interactions that includes the 27 standard pairwise tests used by Elena and Lenski. We then compare the epistatic deviations calculated using the new tests to those obtained from the standard tests, and we use the new tests to extract previously unnoticed features of the fitness landscape. Specifically, we investigate how epistasis depends on the fitness loss associated with deleterious mutations. We also consider tests that provide a new perspective on the relative "mixing ability" of different mutations. Here, the mixing ability of any given mutation specifies whether its epistatic interactions with a set of other mutations tend to be positive or negative. Finally, we describe the geometry of the overall fitness landscape by focusing on the three-locus subsystems whose shapes correspond to the triangulations of the four genotopes shown in Figure 1, panels b-d. We also discuss how this geometric formulation reflects the underlying set of gene interactions.

## Results

### Markov basis of the interaction space

Our first point is that the genotype space in Table 1 allows many more tests of epistasis than the 27 standard tests performed by Elena and Lenski [2]. Notice that the standard test *w·ar - a·r *compares two genotypes having zero and two mutations with two others each having one mutation. The minimal Markov basis (see Methods for a mathematical definition) of the space of tables of the type displayed in Table 1 reveals an additional 216 non-standard tests of the following two sorts. First, there are 108 "double-double" tests that compare two double mutants with two other double mutants, holding the distribution of the mutant alleles constant, for example, *ar·bs *- *as·br*. Second, there are another 108 orthogonal "single-double" tests that compare one single mutant and one double mutant with another single mutant and another double mutant, again holding constant the allele frequencies, for example, *a·br *- *b·ar*. For both types of non-standard tests, the two genotypes on the right-hand side can be regarded as the products of recombination between the two genotypes on the left-hand side. Notice that the same relationship also holds true for the standard tests. 

Experimental biologists (including two authors of this paper) are likely to raise three concerns about these non-standard tests. What biological insights can non-standard tests provide beyond those obtained using standard tests? Are these additional tests independent of the standard tests? What computational tools are available to perform such tests on other datasets?

As we show in the sections that follow, the non-standard tests are potentially useful in at least three respects.  First, they allow one to focus attention on features of epistasis that are not quantifiable by the standard tests.  For example, we perform non-standard tests of the "single-double" type to explore whether some mutations are better overall mixers than others.  Second, non-standard tests span greater genetic distances than do pairwise tests, allowing more powerful analyses of the structure of fitness landscapes.  For example, we use the "double-double" tests to test curvature at genetic distances of four, whereas standard tests allow curvature to be examined only at distances of two.  Third, non-standard tests are an integral part of the complete geometric description of a fitness landscape.  While this high-dimensional geometry is abstract and even foreign, we describe how biological features of gene interactions, such as mixing ability, are embedded in the geometric shape of the landscape.    

The non-standard tests are not independent, in a statistical sense, of the pairwise tests or of one another because all the tests are calculated from the same underlying data.  Nonetheless, this complication can be addressed by employing appropriate statistical methods (Tukey's jackknife, Bonferroni correction, etc.) to ensure that significance levels reflect the data structure.  Regarding the availability of computational tools, we provide references to programs that automate the calculations of the Markov basis and perform the triangulations necessary to describe the geometry of landscapes, and these tools can be applied to other datasets.  In supplementary material, we illustrate the use of these computational tools and show their output (see Additional files 1-5).

### Comparing standard and non-standard epistasis terms overall

An important feature of the standard tests is that the sign of epistasis, either positive or negative, is always expressed in reference to the same wild-type strain. The key result reported by Elena and Lenski, based on the standard tests, was that there were many significant epistatic deviations in both directions, in contrast to one hypothesis that predicted negative epistasis should be the general rule [17]. At first glance, the non-standard tests described above would not seem to allow this prediction to be tested, because the sign of the difference is arbitrary; that is, the labels that put one pair of mutants on one side of the equation, and not on the other side, are arbitrary. However, the biological interest in epistasis is often framed in terms of deleterious mutations, and Elena and Lenski ensured that they had a high-fitness wild-type strain by using one that had evolved for 10,000 generations in the exact same environment employed for measuring the relative fitness of all the genotypes in their study. In the same vein, we can anchor all 216 non-standard equations simply by ensuring that the genotype with the highest fitness (of the four used in any given calculation) appears on the left-hand side of the equation with positive sign.

**Figure 2:**
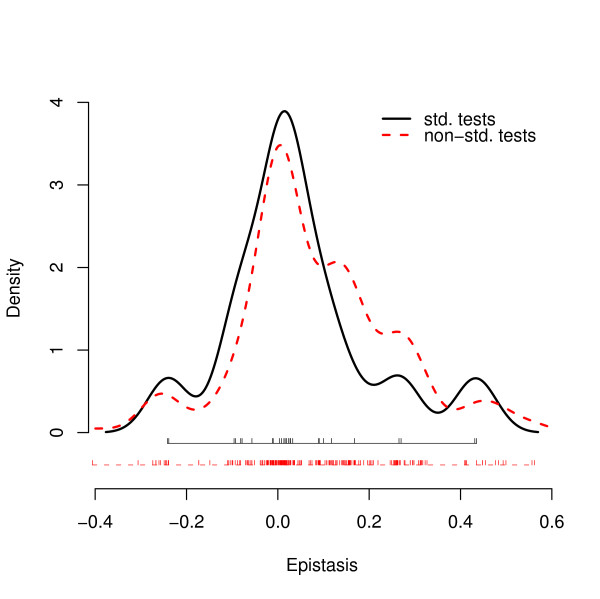
**Standard and non-standard gene interactions**. Displayed are density estimates of gene interactions as measured by the 27 standard tests (solid curve), for example *w·*ar - *a·r*, and by the 216 non-standard tests (dashed curve), for example *ar·bs *- *as·br*. The raw data are shown below the density curves.

Figure 2 shows the distribution of epistatic deviations for all 216 of the non-standard equations (dashed curve) as well as the 27 standard equations (solid curve). The non-standard tests support the inference of Elena and Lenski, based on the standard tests, that many epistatic deviations are positive and many others negative. In other words, one directional form does not predominate to the exclusion of the others. To the eye, it would appear that the non-standard form is more skewed toward positive epistasis. If so, that difference would be interesting because the non-standard tests include genotype sets in which the maximal fitness is usually below the high-fitness wild-type strain. Previous research has shown that compensatory mutations, in which some mutations are conditionally beneficial only in the presence of certain otherwise deleterious mutations, are biologically important [32-34]. Compensatory mutations contribute to positive epistasis and, moreover, they become more important farther away from a local fitness peak [33-36]. In the context of the *E. coli *data that we are analyzing, we predict that epistatic deviations tend to more positive values when the component mutations have more deleterious effects (thus corresponding to greater distances from the local peak).

To test this prediction for the fitness peak at the wild-type, we calculated for each standard test of epistasis (such as *w·ar *- *a·r*) the average fitness decrement Δ = 1 - (*a *+ *r*)/2 of the two single mutants relative to the wild-type. We then plot the epistasis values as a function of Δ for all 27 equations. In this way, we can test the hypothesis that epistatic deviations tend more toward positive values when mutations in the wild-type background are more deleterious (large Δ) than when mutations are less deleterious (small Δ). The scope of this analysis can be extended substantially by also including the non-standard "double-double" epistatic equations (such as *ar·bs *- *as·br*). We anchored each double-double test with the fittest genotype (say *ar*) on the left-hand side, and calculated the average fitness decrement, Δ = *ar *- (*as *+ *br*)/2, of the two genotypes that appear on the right-hand side of the equation. These tests ask more generally for the relationship between epistasis and the average fitness loss relative to any local fitness peak rather than only the one centred on the wild-type. Moreover, these non-standard tests may have greater power because their "reach" extends over mutational distances of four, rather than the two allowed by standard tests. (We have excluded the non-standard single-double tests from this analysis, because in those equations each genotype has different Hamming distances to the two genotypes on the opposite side of the difference equation. This heterogeneity prohibits direct comparisons with the other tests).

**Figure 3:**
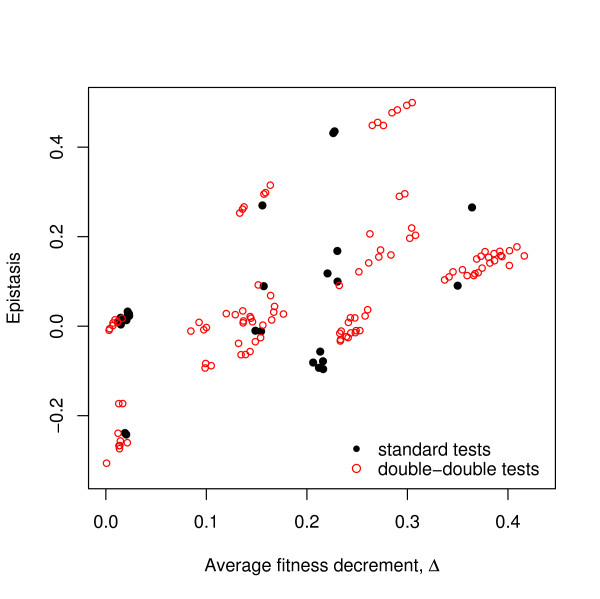
**Epistasis correlates with relative fitness loss**. For each standard test (filled black circles), for example *w·ar *- *a·r*, and each double-double test (open red circles), for example *ar·bs *- *as·br*, anchored with the fittest type on the left-hand side of the equation, the value of the test (*epistasis*) is plotted versus the average fitness decrement (Δ) associated with the two deleterious two-point mutations that define the genotypes on the right hand side of the equation. Epistasis tends toward more positive values in the context of more deleterious mutations. The significance of this correlation was robust to different assumptions about the independence of the data, as described in the text.

Figure 3 shows the resulting relationship between the average fitness decrement associated with component mutations and the value of the epistatic deviation. For both standard and non-standard tests, there is a strong relationship in the predicted direction, such that epistatic interactions tend to be more positive when the component genotypes are less fit, and more negative when those genotypes are fitter. This trend is marginally significant for the 27 standard tests (*r *= 0.433, 25 d.f., *p *= 0.012), but highly significant for the larger set of 108 double-double tests (*r *= 0.597, 106 d.f., *p *<10^-11^), if each of the deviations is viewed as independent. However, these values all rest on 37 genotypes, whose fitness values were estimated with error (albeit with replication), and hence the errors are not independent for those epistatic terms that share a genotype. To take this complication into account, we performed Tukey's jackknife test [37]. Even so, the association remains strongly significant for both the standard (mean *r *= 0.440, *t*_*s *_= 3.761, 26 d.f., one-tailed *p *= 0.0004) and non-standard tests (mean *r *= 0.578, *t*_*s *_= 3.672, 26 d.f., one-tailed *p *= 0.0005). It is important to understand that this inference concerns the relationship between average fitness decrements and the form of epistasis around any local peak within the particular landscape represented by these 37 genotypes. In other words, it is an inference about a restricted region of the complete *E. coli *genotypic space.

If we want to make the same type of inference about the complete genotype space, rather than the specific subset sampled by Elena and Lenski, we can apply a similar, but not identical, test. More precisely, we want to investigate the correlation between average fitness decrease and epistasis among any single and double mutants of the *E. coli *genome. In that case, the unit of independent observation becomes the nine single mutations, and the resulting inference concerns the landscape of all possible double mutations derived from the same parental strain. We again applied Tukey's jackknife, this time eliminating in turn all tests containing a particular mutation in any of the four double mutants, rather than eliminating a particular genotype as before. In this case, the association is only marginally significant (mean *r *= 0.572, *t*_*s *_= 1.363, 8 d.f., one-tailed *p *= 0.105 for the standard tests, and mean *r *= 0.457, *t*_*s *_= 1.780, 8 d.f., one-tailed *p *= 0.056 for the non-standard tests), but still supports the trend for increasingly positive epistasis with increasing average fitness loss. Given that these tests aim to infer a complex genome-wide relationship between average mutational effects and the form of epistasis from a small sample of mutations, it is encouraging to find such a strong trend.

We present these alternative analyses to emphasize the subtly different hypotheses that can be addressed by using our mathematical approach. Comparing the last two analyses suggests that individual mutations might have pervasive effects on the shape of the local landscape. While pervasive effects of certain mutations can make it more difficult to test broader generalizations, the precise nature of these pervasive effects is of biological interest. In the next section, we follow the same general approach, but focusing on a different set of epistatic interactions, to examine differences between individual mutations in greater detail.

### Some non-standard tests reveal differences in mixing ability

In this section, we use non-standard tests of the "single-double" type to explore a particular aspect of the fitness landscape, specifically whether certain mutations are better mixers than others. The mixing ability of any particular mutation indicates whether its epistatic interactions with other mutations tend to be positive or negative. We can then measure the relative mixing ability of two mutations by holding constant the identity of other mutations with which the two of interest are mixed. Consider the polynomial *a·br *- *b·ar*. This test asks, in effect, whether mutation *a *or *b *mixes better with a third mutation *r*. An individual test of this form might be interesting when one has specific knowledge about the identity of the three mutated genes and the position of their products in a metabolic pathway, for example. By contrast, Elena and Lenski emphasized the statistical properties of epistatic interactions. In this context, we can examine related sets of these single-double equations to ask whether one mutation is a better mixer than another in the context of the sample of mutations with which they were each tested.

Any pair of mutations belonging to the same set of three ({*a*, *b*, *c*}, {*r*, *s*, *t*}, or {*x*, *y*, *z*}) was tested with the exact same set of six mutations belonging to the other two sets. For example, the following six equations examine the relative mixing ability of "focal mutations" *a *and *b *with respect to "tester mutations" *r*, *s*, *t*, *x*, *y*, and *z*: *a·br *- *b·ar*; *a·bs *- *b·as*; *a·bt *- *b·at*; *a·bx *- *b·ax*; *a·by *- *b·ay*; and *a·bz *- *b·az*. All in all, there are nine groups of six equations each that compare the general mixing abilities of two focal mutations from the same set: *a *versus *b*, *a *versus *c*, *b *versus *c*, *r *versus *s*, *r *versus *t*, *s *versus *t*, *x *versus *y*, *x *versus *z*, and *y *versus *z*. There are another 18 groups of three equations each that compare the mixing abilities of two focal mutations from different sets. (Nine more groups of this type are redundant, in the sense that their values are determined by the first 18 groups.)

**Figure 4:**
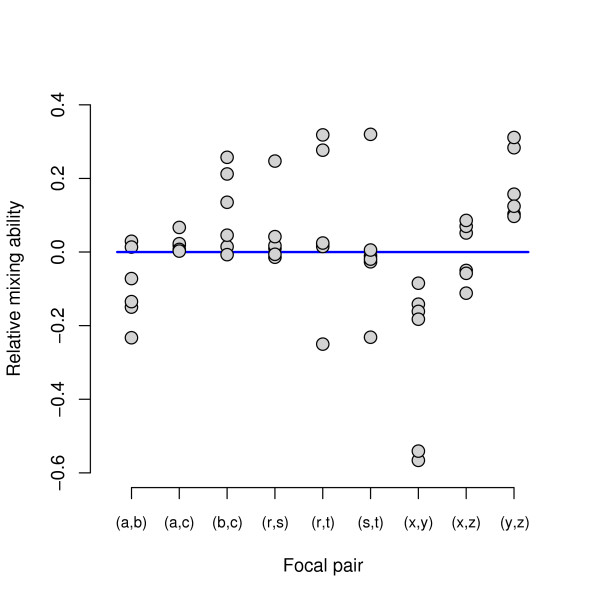
**Mixing ability of mutations**. For each focal pair of mutations (*a*, *b*), ..., (*y*, *z*), six tests of the sort *a·br *- *b·ar *were performed in order to test the relative mixing ability of component mutations *a *and *b *relative to a tester mutation *r*. A small amount of jitter has been added to the vertical coordinate of each point in order to facilitate visualization. Mixing abilities vary considerably between mutations, with the (*y*, *z*) comparison (last column) revealing the most significant difference in favour of *y *as the superior mixer to *z*.

In our analysis, we focus on the nine comparisons of mixing ability that each involves six tester mutations, because these provide more statistical power that might reveal differences between focal mutations. Figure 4 summarizes these nine comparisons. Six points are plotted above each pair of focal mutations, corresponding to the six different tester mutations with which each one was combined. A value above zero indicates that the first-listed mutation in the focal pair was the better mixer. For example, for the first pair (*a*, *b*) of focal mutations, *a *was the better mixer with two tester mutations whereas *b *mixed better with four others. For each focal pair, we performed a *t*-test to ask whether the average epistatic deviation was significantly different from zero. The focal pairs (*x*, *y*) and (*y*, *z*) were both significant (*p *≤ 0.027), with *y *being the better mixer in both cases. Two other focal pairs, (*a*, *b*) and (*b*, *c*), were marginally non-significant with *p *= 0.083 and *p *= 0.068, respectively, and *b *was the better mixer in both of these cases. The (*y*, *z*) comparison survives even a stringent Bonferroni correction that accounts for the multiplicity of related tests (*p *= 0.0054·9 < 0.05). Therefore, we can conclude that mutations are variable in their general mixing ability. The molecular identities of mutations were not identified by Elena and Lenski [2], and so we cannot say anything about the potential physiological bases for the observed differences in mixing ability. However, future studies of epistatic interactions might systematically compare mixing ability between different classes of mutations, such as those affecting protein structures and regulatory domains.

### Geometry of the fitness landscape

Our final set of results is concerned with the geometric shape of the fitness landscape. Since fitness landscapes are high-dimensional and complicated objects, it is desirable to classify them into a finite set of distinct shapes; with the general idea that fitness landscapes with the same shape are likely to share biological properties. This approach generalizes the classification of bi-allelic two-locus landscapes into those with positive epistasis versus those with negative epistasis. This appealing binary classification has been linked, for example, to the advantage of sex, but it does not extend to higher-dimensional genotype spaces. We present here a notion of the shape of a fitness landscapes for any genotypic space. This concept is intimately related to the interaction tests discussed so far, because the shape is determined by a certain subset of the gene interactions that includes the Markov basis. Thus, the proposed classification of landscapes into shapes can be regarded as a formal summary of all the various standard and non-standard tests.

The fitness landscape studied in this paper consists of the 37 *E. coli* genotypes and their fitness values as shown in Table 2.  The genotope is the set of all possible allele frequencies that can be realized by any population on these genotypes.  It is a nine-dimensional figure with 37 vertices, and it contains all the three-dimensional genotopes shown in Figure 1.  By the shape of the fitness landscape we mean the triangulation of the genotope that is induced by the fitness values (see Methods for details).  Since most of us have trouble visualizing and interpreting nine-dimensional objects, we therefore study the shape by analyzing its restrictions to genotypes on two and three of the nine loci.  In so doing, we aim to illustrate how certain features of the fitness landscape - in particular, differences in mixing ability revealed by the non-standard tests - are reflected in the geometry of the fitness landscape.

Consider the bi-allelic two-locus system with genotypes *w, a, r*, and *ar*.  Its genotope is the unit square in (*a, r*)-space, and a generic fitness landscape has exactly one of two possible shapes corresponding to either negative or positive epistasis, i.e., to *w  ar* - *a r* being either negative or positive.  Negative epistasis induces the triangulation of the square consisting of the two triangles {*w, a, r*} and {*a, r, ar*}, whereas positive epistasis results in the other possible triangulation with {*w, a, ar*} and {*w, r, ar*}.

For larger genetic systems, the role of the triangles is played by simplices.  The shape of the present fitness landscape on 37 genotypes is a triangulation of the genotope into 362 nine-dimensional simplices (see Additional file 5).  The following analysis of the geometry of this space is based on the general framework developed elsewhere [1], as applied to the specific experimental design that generated the *E. coli* fitness landscape investigated in this paper.  The general idea is to investigate and describe the interaction space.  This space has finite dimension, but infinitely many elements, and each element of this space represents one interaction.  There are many different ways of extracting potentially interesting subsets of the interaction space.  We suggest looking at the circuits, which generate the space but are not linearly independent.  The signs of the circuits determine the triangulation of the genotope and thus the shape of the fitness landscape.  However, the number of circuits grows fast with the size of the fitness landscape; the present *E. coli* landscape has 772,731 circuits.  Therefore, we restrict our attention to the much smaller subset provided by the Markov basis.  The circuits and the Markov basis provide a natural generalization of the concept of pairwise epistasis.

**Figure 5:**
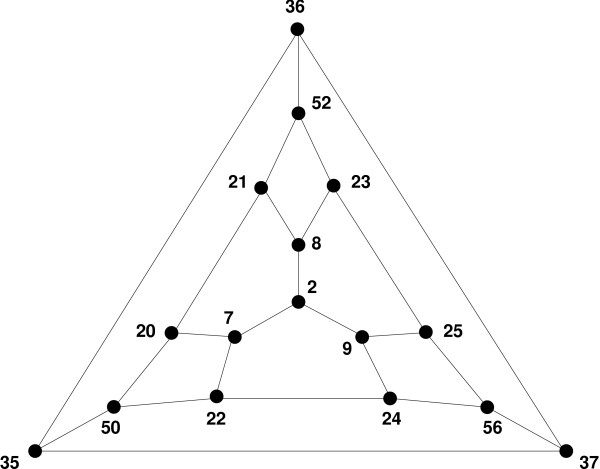
**The 16 shapes of fitness landscapes of genotope (b)**. In the graph, each vertex represents one of the 16 possible geometric shapes of a fitness landscape on the truncated three-locus system that corresponds to genotope (b) in Figure 1. The shapes are determined by a collection of different tests for gene interactions. Two shapes are connected by an edge if they differ only by the sign of a single test. The labelling of the vertices follows [1, Table 5.1].

The three-locus subsystems that occur in this dataset are represented in Figure 1b–d by their genotopes. Notice that type (d) cannot be further subdivided. By contrast, type (c) has six triangulations and type (b) has 16 triangulations. We focus on type (b) in order to show how the different shapes are related. The signs of a total of nine circuits (or tests) determine the geometry, including three standard tests like *w·ar *- *a·r*, three non-standard single-double tests, and three cubic tests that are not part of the minimal Markov basis. In Figure 5, each of the 16 shapes is represented by a vertex in the graph and labelled by an integer. The labels refer to the 74 shapes of the 3-cube (see Table 5.1 in [1] for the complete list), 16 of which occur here as the shapes of the type (b) genotype space. Two shapes in the graph are connected by an edge if they differ only by the sign of a single test. For example, shapes 36 and 37 differ by the sign of *r·ax *- *x·ar*. Thus, the graph represents the 16 possible shapes of a fitness landscape over the three-locus genotype space consisting of seven genotypes (all but the triple mutant). This graph provides the basis for statistical inference about the three-way interactions in the given fitness landscape. We find the following shapes among all 27 of the three-dimensional genotopes of type (b) that appear as subsets of the complete dataset: shape 56 (frequency: 6), 7 (5), 25 (4), 8 (3), 52 (3), 21 (2), 23 (2), 2 (1), and 20 (1). A closer inspection of this shape distribution reveals many deviations from linearity in the fitness landscape, but no single dominating shape. This result therefore confirms and extends the earlier finding of Elena and Lenski [2] about the commonness of deviations from linearity.

The analogous graph of possible shapes for the three-locus subsystem corresponding to the triangular prism (Figure 1c) is a hexagon. In fact, that hexagon occurs three times as a sub-graph in the graph for type (b) illustrated in Figure 5, because the genotope (c) is contained within the genotope (b) in three different orientations. Intuitively, any three-locus genotype space lacking a double and the triple mutant can be regarded as a subspace of the space that only lacks the triple mutant. The six triangulations of genotope (c) are represented in Figure 5 by the three hexagons with vertices {35, 36, 52, 21, 20, 50} (upper left), {36, 37, 56, 25, 23, 52} (upper right), and {37, 35, 50, 22, 24, 56} (bottom), respectively, that are related by symmetry.

The shape of fitness landscapes on type (c) spaces, such as {*w*, *a*, *b*, *r*, *ar*, *br*}, is determined by the signs of the three tests *w·ar *- *a·r*, *w·br *- *b·r*, and *b·ar *- *a·br*. Therefore, the shape summarizes the information about the standard pairwise interactions between mutations *a *and *r *and between *b *and *r*, as well as the relative mixing ability of *a *and *b *with respect to *r*. For example, five of the six subsystems of type (c) involving mutations *y *and *z *have shape 21, while the subspace {*w*, *y*, *z*, *b*, *by*, *bz*} has shape 52. Shape 21 is defined by a negative sign for the second of the three tests above and a positive sign for the other two, whereas shape 52 is defined by positive signs for all three tests. Hence, these shapes reflect positive epistasis involving *y *(6/6 tests), positive epistasis involving *z *(5/6 tests), and superior mixing ability of *y *over *z *(6/6 tests). As such, the geometric shapes provide more details about the form of mutational interactions than the average values of the tests analyzed in the previous section. On the other hand, these shapes summarize the data by classifying continuous fitness values into discrete shape classes which reflect the sign pattern of the interaction tests.

## Discussion and Conclusion

Epistasis occurs whenever mutations interact non-linearly with one another, and it represents a major challenge in describing the mathematical structure of real fitness landscapes. With epistatic interactions, the combined effect of two or more mutations on fitness may be greater than, less than, or opposite in sign to expectations obtained by combining their separate effects. A growing body of empirical research indicates that epistasis is very common in nature [2,11,21,23,26-34], [39,40]. However, a complete mathematical description of epistatic interactions has not been forthcoming for any system because the forms of epistasis appear to be diverse, idiosyncratic, and hence complex.

To date, two different aspects of epistasis have served as summary statistics of these interactions. First, studies have used the overall directional curvature of mean fitness as a function of the number of random mutations introduced into the genome of some wild-type organism [2,27-31]. An older variant of this approach uses the time during which mutations have accumulated in a population subjected to severe bottlenecks as a proxy for estimating the number of mutations [41-44]. In any case, the absence of overall directional curvature does not distinguish between two biological scenarios: (i) most mutations have independent effects such that there is very little epistasis; and (ii) epistasis is common but interactions between mutations are diverse in their directional effects, thereby obscuring overall average curvature [2]. These two scenarios make different predictions about the evolution of a population on a fitness landscape that may confound efforts to understand, for example, the evolution of sexual reproduction [18,20]. The second summary of epistasis considers the statistical distribution of a particular class of epistatic interactions, typically pairwise [45]. For example, Elena and Lenski [2] found that average fitness as a function of mutation number did not deviate significantly from log-linearity, which might suggest that epistasis is rare. But in a second experiment, they showed that significant pairwise interactions were common, although some were positive and others negative, such that there was no clear trend with respect to overall directional curvature.

The objective of this paper is to introduce biologists to a new mathematical framework for characterizing epistatic interactions between mutations, which goes beyond both overall directional curvature and pairwise interactions by providing a complete geometrical description of the epistatic interactions that define an empirically determined fitness landscape. To that end, we have re-analyzed the dataset from the pairwise experimental design performed by Elena and Lenski [2] using this new approach. In addition to providing an overall geometric description, various biologically motivated tests about the forms of epistasis are embedded in this framework. In particular, the geometric framework allows not only tests of the standard pairwise interactions but also non-pairwise tests that gave new insights into (i) the relationship between the form of epistasis and the individual mutational effects, and (ii) variation between mutations in their mixing ability with other mutations.

The fitness landscape that we analyzed comprises 37 genotypes of *E. coli *that were constructed by introducing nine mutations into a wild-type strain and constructing a restricted set of 27 double mutants. Despite the absence of any triple or other higher-order mutants in the dataset, our analysis reveals complex new epistatic interactions, beyond the pairwise interactions reported previously. First, our analysis confirms and extends Elena and Lenski's inference that the fitness landscape is complex, such that an overall measure of curvature obscures a complex admixture of interaction types, some with positive and others with negative effects on fitness (Figure 2). Second, we calculated the set of non-standard interactions that contrast two double mutants with two other double mutants, while holding all of the component alleles constant. In doing so, we found a strong correlation between the average fitness decrement associated with mutations and the resulting form of epistasis, such that epistatic deviations tend toward more positive fitness effects when the component mutations are more deleterious (Figure 3). This finding also led us to re-examine interactions based on the standard pairwise tests for evidence of this relationship, and the same trend was evident. This correlation is consistent with previous studies showing that compensatory mutations, which contribute to positive epistasis, become more important as one moves farther away from a local fitness peak [33-36]. More generally, this association emphasizes that any particular epistatic interaction is a local feature in the fitness landscape, and this association identifies one source of variation among local features.

Third, we show that individual mutations contribute in different ways to the complex admixture of epistatic interactions. In particular, we found that some mutations are better mixers than other mutations (Figure 4). That is, double mutants that include mutations that are relatively "good mixers" tend to be more fit than double mutants that harbour "bad mixers", even when the identity of their partner mutations is held constant. Although our analyses have not specifically addressed the evolution of sexual recombination, we suggest that appropriately designed tests of mixing ability may provide valuable insights into this area. A key difference between asexual and sexual reproduction is that the fate of a particular mutation is tied to its fitness effect in the genetic background where it arose in the case of asexual reproduction, while a mutation's fate in a sexual population depends on its effects over many backgrounds [46,47]. Finally, we determine the overall geometric shape of the fitness landscape, which summarizes all the biologically interesting features described above (Figure 5).

In closing, we would like to raise an issue related to experimental design, one that requires attention when planning studies that might employ these new approaches to testing epistatic interactions and describing fitness landscapes. In their paper, Elena and Lenski [2] viewed it as problematic that the same mutations were used in multiple genotypes, because this compromised the statistical independence of some of their observations. They addressed this problem by applying Bonferroni corrections to their statistical tests, but they could instead have made 27 double-mutant genotypes with no overlap in the constituent mutations. Fortunately, they did not do so because, if they had, it would not have been possible to apply the mathematical approaches we have used to analyze the epistatic interactions and the shape of the underlying fitness landscape. Thus, minimizing the mutational overlap between genetic constructs may simplify statistical analyses, but it also constrains the analysis of more complex forms of epistasis. In particular, the existence of shared mutations allowed us to examine genotypes that spanned mutational distances of 3 (single-double tests) and 4 (double-double tests), which were essential for achieving the new inferences outlined in this study. Therefore, we recommend that future studies of epistatic interactions include many genotypes that share mutations in order to explore the geometry of the fitness landscape more fully. Of course, the inclusion of triple mutants and other higher-order genotypes can extend the reach of our geometric approach but, even then, the reach will be greater still if two or more sets of higher-order genotypes share some mutations.

## Methods

### Bacterial experiments

Our analyses use the data obtained from the second of two experiments reported by Elena and Lenski [2]. Details of genotype construction and competition assays are presented there. Briefly, they constructed 9 genotypes, all from the same initial strain, that each had a single mutation caused by the insertion of one of three Tn*10 *mini-transposons carrying different antibiotic-resistance markers. Other work indicated that the site of an insertion is largely responsible for a mutation's effect on fitness [48]. They then constructed 27 genotypes each carrying two of these mutations. Nine of the 36 possible double mutants were not constructed owing to restrictions based on having only three resistance markers, and no triple mutants were constructed in that experiment. The fitness of each of these 9 single mutants and 27 double mutants was measured in competition with the wild-type strain. Fitness measures the growth rate of a genotype realized during competition with the wild-type, and it is also expressed relative to the realized growth rate of the wild-type. Each pair-wise competition was replicated 10-fold and, in our analyses, we have used the median of the 10 estimates as the fitness value for each genotype. The fitness of the wild-type strain was set to 1.

### Mathematical framework

Our analysis is based on the mathematical framework presented by Beerenwinkel et al. [1]. The dataset analyzed here induces the genotype space *G *⊆ {0,1}^9 ^of size 37 consisting of the wild-type strain *w *= 000000000, the nine single mutants *a *= 100000000, ..., *z *= 000000001, and the subset of 27 double mutants *ar *= 100100000, ..., *tz *= 000001001 (Table 1). We denote by Δ_*G *_⊂ **R**^37 ^the set of all populations (genotype frequencies) on *G*. The genotope Π_*G *_⊂ **R**^9 ^is the set of all allele frequencies that can be realized by populations on *G*. The genotope is a nine-dimensional polytope, defined as the convex hull of the 37 genotypes, i.e., as the set of all convex combinations ∑g∈Gλg⋅g
 MathType@MTEF@5@5@+=feaafiart1ev1aaatCvAUfKttLearuWrP9MDH5MBPbIqV92AaeXatLxBI9gBaebbnrfifHhDYfgasaacH8akY=wiFfYdH8Gipec8Eeeu0xXdbba9frFj0=OqFfea0dXdd9vqai=hGuQ8kuc9pgc9s8qqaq=dirpe0xb9q8qiLsFr0=vr0=vr0dc8meaabaqaciaacaGaaeqabaqabeGadaaakeaadaaeqbqaaGGaciab=T7aSnaaBaaaleaacqWGNbWzaeqaaOGaeyyXICTaem4zaCgaleaacqWGNbWzcqGHiiIZcqWGhbWraeqaniabggHiLdaaaa@39AA@, with *λ*_*g *_≥ 0 and ∑g∈Gλg=1
 MathType@MTEF@5@5@+=feaafiart1ev1aaatCvAUfKttLearuWrP9MDH5MBPbIqV92AaeXatLxBI9gBaebbnrfifHhDYfgasaacH8akY=wiFfYdH8Gipec8Eeeu0xXdbba9frFj0=OqFfea0dXdd9vqai=hGuQ8kuc9pgc9s8qqaq=dirpe0xb9q8qiLsFr0=vr0=vr0dc8meaabaqaciaacaGaaeqabaqabeGadaaakeaadaaeqaqaaGGaciab=T7aSnaaBaaaleaacqWGNbWzaeqaaaqaaiabdEgaNjabgIGiolabdEeahbqab0GaeyyeIuoakiabg2da9iabigdaXaaa@37B4@). Figure 1 shows the genotopes of all three-locus subsystems induced by *G*, all of which are contained in Π_*G *_.

There is a natural mapping ρ : Δ_*G *_→ Π_*G *_that assigns to each population its allele frequency spectrum. The kernel of this map defines the interaction space (see Section 3 in [1]). The elements of the interaction space measure the gene interactions that underlie the given fitness landscape on *G*. They include both the standard and non-standard tests used in our analyses.

The specific experimental design that was used to generate the present fitness dataset allows us to represent the genotypes in the matrix displayed in Table 1. The entries of this table are interpreted as variables. If we regard Table 1, hypothetically, as a contingency table of genotype counts, then taking row sums (or, equivalently, column sums) corresponds precisely to evaluating the mapping *ρ*. The kernel of *ρ *is the space of integer tables of the same format with zero margins (row sums). A canonical set of generators for this kernel is the minimal Markov basis [49], which consists of polynomials in the unknown variables, one for each genotype. These polynomials correspond to the minimal generators of the toric ideal associated with *ρ *(see [51] for an introduction to toric ideals). The Markov basis represents the fundamental gene interactions that underlie the given fitness landscape. It consists of the 27 standard tests and the 216 non-standard tests that are described in the Results section and listed in supplementary material ( see Additional file 4).

A simplex is an *n*-dimensional analogue of a triangle. Formally, a simplex can be defined as the convex hull of a set of (*n *+ 1) affinely independent points in **R**^*n *^[50]. This means that the (*n *+ 1) points span an affine space of dimension *n*. For example, Figure 1d shows a 3-simplex (known as a tetrahedron), which is the convex hull of the points *w*, *a*, *b*, and *c*. By a triangulation of a polytope we mean its decomposition into a set of simplices. A fitness landscape on *G *gives rise to a triangulation of the genotope Π_*G *_(see Section 4 in [1]). Thus, the shapes of fitness landscapes on a genotype space *G *are defined as the induced triangulations of its genotope. These triangulations are determined by the initial ideal of the toric ideal associated with *ρ*. The derivation of the triangulation from the initial ideal is explained in [[51], Chapter 8]. The specific triangulation induced by the data in Table 2 consists of 362 simplices and is provided as supplementary material (see Additional file 5). In Figure 5, all possible shapes are displayed for the three-locus system that corresponds to the genotope shown in Figure 1b. In fact, the shapes correspond to the vertices of the three-dimensional secondary polytope of this genotope.

### Computational methods

The Markov basis can always be derived from the genotype space (the set of measured genotypes) by algebraic computations.  However, there is no simple recipe for writing out the Markov basis.  We computed the Markov basis independently with the computer algebra systems Macaulay 2 [52] and Singular [53].  The triangulation was computed independently with Macaulay 2 and 4ti2 [54].  All statistical computations were performed in R [55].  In our supplementary materials, we illustrate the use of the Macaulay 2 program for calculating the Markov basis and triangulation of a dataset [see Additional files 1-3].

## Authors' contributions

N.B., L.P., and B.S. are responsible for the development of the mathematical theory. S.F.E. and R.E.L. designed and performed the experiments with bacteria. N.B. and R.E.L. formulated the specific analyses reported here and wrote the paper. All the authors contributed to editing the paper and approve of its final form.

## Supplementary Material

Additional file 1 Instructions for calculating the Markov basis and triangulation of a dataset with Macaulay 2. Click here for file

Additional file 2 Macaulay 2 file for computing Markov bases of interaction spaces and triangulations of genotopes. (This program requires Macaulay 2 version 0.0.95 to be installed. See additional file 1 for instructions.) Click here for file

Additional file 3 Examples of fitness landscape files to be processed with the program fitness.m2 (additional file 2). Click here for file

Additional file 4 Minimal Markov basis of the interaction space. Click here for file

Additional file 5 Geometry of the fitness landscape. Click here for file
