# Supplementary material for: Analysis of epistatic interactions and fitness landscapes using a new geometric approach
Source: BMC Evol Biol. 2007 Apr 13;7:60. doi: 10.1186/1471-2148-7-60 (PMC1865543; doi:10.1186/1471-2148-7-60)
Supplement: Additional file 1 — Instructions for calculating the Markov basis and triangulation of a dataset with Macaulay 2. [file 1471-2148-7-60-S1.pdf]

# Computing Markov bases and triangulations with *Macaulay 2*

(Additional file 1 of Beerenwinkel *et al.*, **Analysis of epistatic interactions and fitness landscapes using a new geometric approach**, *BMC Evolutionary Biology)*

This note will guide the reader through using the computational algebra software *Macaulay 2* for computing the Markov basis of a genotope and the triangulation of a fitness landscape.

## 1 Software

*Macaulay 2* is a software system devoted to symbolic computation and computational algebra. It can be run as an interactive shell and it provides a programming language for solving computational algebraic problems. The *Macaulay 2* home page at `http://www.math.uiuc.edu/Macaulay2/` offers free download of the software, documentation, and references. *Macaulay 2* runs under all common operating systems, including Linux, Windows, and Mac OS X.

The program `fitness.m2` is written in *Macaulay 2* language and requires *Macaulay 2* version 0.9.95 to be installed. It contains the function `fitnessShape` which takes as input a fitness landscape and will output the Markov basis of the interaction space and the shape of the fitness landscape, i.e., the triangulation of the genotope induced by the fitness values.

To get started, copy the file `fitness.m2` into your working directory, start *Macaulay 2*, and enter the command

```
i1 : load "fitness.m2"
```

The program `fitnessShape` is now ready to be used.

## 2 Markov bases and triangulations

The fitness landscape is specified in a separate file. For example, let us consider the following fitness landscape on five genotypes that form a bipyramid inside the 3-cube:

| Genotype | Fitness |
|---|---|
| 000 | 0.99 |
| 001 | 0.98 |
| 010 | 0.976576 |
| 100 | 0.98767 |
| 111 | 0.5565 |

In the corresponding fitness landscape file that needs to have the suffix `.fl`, each line contains one genotype followed by its fitness. For example, the data above might be stored in the text file `mydata.fl` with lines

```
000     0.99
001     0.98
010     0.976576
100     0.98767
111     0.5565
```

The corresponding data file for the 37 *E. coli* genotypes is provided in Appendix A.

Now, the function `fitnessShape` takes the filename (without the suffix `.fl`) as an argument. Thus, the command

```
i2 : fitnessShape "mydata"
```

will compute the Markov bases and the triangulation and write the result to the output file `mydata.outm2`. Using

```
i3 : (L, B, S) = fitnessShape "mydata"
```

the different outputs can also be inspected separately. The fitness landscape is stored in `L`,

```
i4 : show L

o4 = index   genotype        fitness
     1       000             0.99
     2       001             0.98
     3       010             0.976576
     4       100             0.98767
     5       111             0.5565
```

The Marbov basis `B` consists of a list of polynomials (here of length 1),

```
i5 : show B

     +-----------------------+
     |                2      |
o5 = |x    x    x    - x    x    |
     | 001 010 100    000 111|
     +-----------------------+
```

Thus, the Markov basis suggests the test

$$f = w_{001}\, w_{010}\, w_{100} - w_{000}^2\, w_{111}$$

for this genotype space.

The shape `S` of the fitness landscape is a list of simplices, each simplex given by the set of its vertices:

```
i6 : show S

     +---------------+
o6 = |001 010 100 111|
     +---------------+
     |000 001 010 100|
     +---------------+
```

Thus, the fitness landscape induces the triangulation of the bipyramid that consists of the two tetraheda

$$\{001, 010, 100, 111\} \quad \text{and} \quad \{000, 001, 010, 100\}.$$

Finally, the three objects can also be computed separately,

```
i7 : L = readLandscape "mydata";  show L

i8 : B = markovBasis L;  show(B,3)

i9 : S = fitnessShape(L,B);  show S
```

# 3 Limitations

In the current implementation of `fitnessShape` fitness values are internally represented as integers in order to be processed by various *Macaulay 2* commands. Consequently, real fitness values are multiplied by a large number and the result is rounded to the nearest integer. This procedure can lead to inaccurate results if the fitness values are very close to each other and the multiplier is not large enough (see file `fitness.m2`).

Similarly, non-generic polyhedral subdivisions will be represented as triangulations by reverse lexicographic tie breaking in *Macaulay 2*.

## A *E. coli* fitness landscape

| | |
|---|---|
| 000000000 | 0 |
| 100000000 | -0.024753864 |
| 100100000 | -0.010403934 |
| 100010000 | -0.027576768 |
| 100001000 | -0.010555514 |
| 100000100 | -0.018113055 |
| 100000010 | -0.331146444 |
| 100000001 | -0.693747361 |
| 010000000 | -0.346017649 |
| 010100000 | -0.036871475 |
| 010010000 | -0.379212737 |
| 010001000 | -0.365643614 |
| 010000100 | -0.246412178 |
| 010000010 | -0.40917197 |
| 010000001 | -0.673932962 |
| 001000000 | -0.025215249 |
| 001100000 | -0.016841017 |
| 001010000 | -0.025266527 |
| 001001000 | -0.026395311 |
| 001000100 | -0.023217451 |
| 001000010 | -0.431552443 |
| 001000001 | -0.730122417 |
| 000100000 | -0.019182819 |
| 000100100 | -0.331494645 |
| 000100010 | -0.011667805 |
| 000100001 | -0.645405185 |
| 000010000 | -0.016586804 |
| 000010100 | -0.323516525 |
| 000010010 | -0.014200349 |
| 000010001 | -0.71396232 |
| 000001000 | -0.005012542 |
| 000001100 | -0.018214888 |
| 000001010 | -0.387207792 |
| 000001001 | -0.676781826 |
| 000000100 | -0.021836695 |
| 000000010 | -0.572878348 |
| 000000001 | -0.523404406 |

## B Complementary software

Other sotware systems that can be useful for computing Markov bases and triangulations include *Singular* (`http://www.singular.uni-kl.de/`), *4ti2* (`http://www.4ti2.de/`), and *TOPCOM* (`http://www.uni-bayreuth.de/departments/wirtschaftsmathematik/rambau/TOPCOM/`).
